# Impact of COVID-19 pandemic on major depressive disorder in acute psychiatric inpatients

**DOI:** 10.3389/fpsyg.2023.1181832

**Published:** 2023-05-25

**Authors:** Claudio Brasso, Marta Cisotto, Elisa Del Favero, Benedetta Giordano, Vincenzo Villari, Paola Rocca

**Affiliations:** ^1^Dipartimento di Neuroscienze “Rita Levi Montalcini, ” Università degli Studi di Torino, Turin, Italy; ^2^Dipartimento di Neuroscienze e Salute Mentale, A.O.U. Città della Salute e della Scienza di Torino, Turin, Italy

**Keywords:** major depressive episode, psychotic features, suicidal ideation, hospitalization, antidepressant increase, augmentation strategies

## Abstract

**Introduction:**

The spread of the coronavirus disease 2019 (COVID-19) pandemic and the subsequent restrictions significantly affected mental health, especially major depressive disorder (MDD) whose incidence increased by 27.6% in 2020, after the COVID-19 outbreak. Few studies focused on the impact of the pandemic on the clinical characteristics of outpatients with MDD and even fewer on inpatients admitted for a major depressive episode (MDE). We aimed to compare the characteristics of MDD of two groups of patients admitted for an MDE before and after the pandemic outbreak and to investigate which variables are significantly related to post-lockdown hospitalizations.

**Methods:**

This retrospective study included 314 patients with MDD hospitalized from January 2018 to December 2021 for an MDE (DSM-5) before (*n* = 154) and after (*n* = 160) the Italian lockdown (9th of March 2020). We compared patients' sociodemographic and clinical characteristics. The characteristics significantly different between the two groups were included in a logistic regression to identify the factors more strictly associated with post-lockdown hospitalizations.

**Results:**

During post-lockdown hospitalization, we found a higher rate of severe MDE (33 patients, 21.4%, in the pre-lockdown and 55 patients, 34.4%, in the post), MDE with psychotic features (3 patients, 2.0%, in the pre-lockdown and 11 patients, 6.9%, in the post-lockdown), and suicidal ideation (42, 27.3%, in the pre-lockdown and 67, 41.9%, in the post-lockdown), with a lower proportion of patients followed by psychiatric services before admission (106 patients, 68.8%, in the pre-lockdown and 90 patients, 56.3%, in the post-lockdown) and a higher percentage of them in treatment with psychotherapy (18 patients, 11.7% in the pre-lockdown and 32, 20.0%, in the post-lockdown) and more frequent increase of the antidepressant dosage (16 patients, 10.4% in the pre-lockdown and 32 patients, 20.0% in the post-lockdown) and adoption of augmentation strategies (13 patients, 8.4%, in the pre-lockdown and 26 patients, 16.3%, in the post-lockdown) to treat the MDE. In the regression model, post-lockdown hospitalizations were significantly associated with suicidal ideation (OR = 1.86; *p* = 0.016) and psychotic features (OR = 4.41; *p* = 0.029) at admission, the increase in the antidepressant daily dose (OR = 2.45; *p* = 0.009), and the employment of an augmentation therapy (OR = 2.25; *p* = 0.029).

**Discussion:**

These results showed an association between the COVID-19 pandemic and the occurrence of MDE with more severe clinical features. This might be true also for future calamities, suggesting that in these emergency contexts, patients with MDD would require more attention, resources, and intense treatments with a specific focus on suicide prevention.

## Introduction

The spread of the coronavirus disease 2019 (COVID-19) pandemic and the subsequent restrictions on social contact had a considerable impact on daily life, substantially affecting the psychological wellbeing of the worldwide population (Torales et al., 2020; Chen et al., 2021; Prati and Mancini, 2021) and leading to a “global mental health crisis” (Rahman et al., 2020; UN, 2020). Many studies were carried out to better understand the long-term mental health implications of the COVID-19 pandemic. Most studies found a strong correlation between the COVID-19 pandemic outbreak and the increase in depressive symptoms in the general population (Ettman et al., 2020; Briggs et al., 2021; Jung et al., 2021; Kwong et al., 2021; Martinelli et al., 2021; van den Besselaar et al., 2021; Gigantesco et al., 2022; Lewis et al., 2022; Medda et al., 2022; Rius Ottenheim et al., 2022; Young et al., 2022). According to some Italian national studies, this association was tighter at the beginning of the lockdown (March 2020) arranged to control the spread of the COVID-19 pandemic (Fiorillo et al., 2020; Mazza et al., 2020; Amerio et al., 2021).

Robinson et al. (2022) reviewed the available literature examining changes in different psychiatric symptoms before and during the pandemic in 2020. The authors found an increase in depressive symptoms that tended to be larger and remained significant also in the later stages of the pandemic. More severe depressive symptomatology was found when higher numbers of COVID-19 cases were reported by media and in countries where more stringent governmental measures to prevent the diffusion of the virus were applied (Salanti et al., 2022). Specific groups of vulnerable people also exhibited more severe depressive symptoms, namely, women (Ahmad et al., 2020; Wang et al., 2020; Daly and Robinson, 2021; Shah et al., 2021; Medda et al., 2022), younger people (Pierce et al., 2020; Praticò, 2021; Shah et al., 2021; Brasso et al., 2022), older people (Ishikawa, 2020), students (Barbosa-Camacho et al., 2022; Medda et al., 2022), healthcare professionals (Rossi et al., 2020), people with a higher education level (Moghanibashi-Mansourieh, 2020; Daly and Robinson, 2021), retirees, unemployed, housewives, and people living in smaller housing spaces (ISTAT, 2022).

Studies that focused on the relationship between COVID-19 and mental disorders reported results similar to those summarized above about psychiatric symptoms in the general population. In particular, a large systematic review and meta-analysis found an increase of 27.6% in the prevalence of major depressive disorder (MDD) after the outbreak of the COVID-19 pandemic (COVID-19 Mental Disorders Collaborators, 2021) with a peak of the incidence from March to May 2020 in conjunction with the first restriction measures (Xiong et al., 2020; COVID-19 Mental Disorders Collaborators, 2021). The increase in the prevalence of MDD was higher in young people and women (COVID-19 Mental Disorders Collaborators, 2021). A total of three studies specifically assessed the impact of COVID-19 on the clinical characteristics of patients diagnosed with MDD (Leightley et al., 2021; Concerto et al., 2022; Siddi et al., 2022), but two of them did not find any changes between pre, during, and post-lockdown (Leightley et al., 2021; Siddi et al., 2022), while Concerto et al. (2022) which suggested an association between COVID-19 pandemic, post-traumatic stress disorder-like symptoms, anxiety, and maladaptive coping, in inpatients with MDD. Moreover, a large observational Chinese study analyzed the prevalence of suicidal ideation and suicide plans and attempts in 1,718 patients with stable MDD from October 2020 and October 2021 reporting a high occurrence of at least one of these three suicide-related phenomena (68.04%) (Zhang et al., 2022). Due to the lack of studies on this topic, we decided to focus on the relationships between the COVID-19 pandemic and the clinical characteristics of inpatients hospitalized for a major depressive episode (MDE). Therefore, the aims of this study were to compare the sociodemographic and clinical features of two groups of inpatients with MDD admitted for an MDE before or after the pandemic outbreak and at investigating which variables are more tightly related to post-lockdown hospitalizations.

## Methods

### Study design

This observational retrospective study was performed in the psychiatric units “Servizio Psichiatrico di Diagnosi e Cura” and “Struttura Complessa Psichiatria Universitaria,” both parts of the Department of Neuroscience and Mental Health of the university hospital “Città della Salute e della Scienza di Torino,” Turin, Italy. This university hospital is one of the first health centers in Italy in terms of size and provides advanced care for the treatment of complex clinical conditions, and its catchment area covers the entire Piedmont region. The period of observation of the study was from 1 January 2018 to 31 December 2021.

The inclusion criteria were principal diagnosis at the discharge of MDE in a MDD (DSM-5 criteria), length of hospitalization >48 h, and age >16 years. We included only admissions lasting more than 48 h because in shorter admissions, the collected information was limited. If patients were hospedalized more than once in the period of observation of the study, we considered the last admission only. This choice was made for two reasons: (i) it was the hospitalization in which more information regarding the entire clinical history of the patient was provided and (ii) we wanted to consider the last hospitalization for all patients.

The sample was divided into two groups: patients hospitalized before the first Italian lockdown and patients hospitalized after the lockdown. The Italian lockdown started on 9 March 2020, as established in the *Decreto del Presidente del Consiglio dei Ministri* n.14 on 9 March 2020, published in the “Gazzetta Ufficiale della Repubblica Italiana del Governo Italiano.”

### Data collection

All data concerning the hospitalization included in the study were obtained from an electronic healthcare database. We collected the following variables.

Socio-demographic and care-related variables: age, nationality (Italian, European or extra-European), gender, single or in a stable relationship, level of education (more or <8 years), employment status, presence of a good social and/or familiar network, primary healthcare at a medical practitioner available at the admission, and patient already followed by psychiatric services at the admission.

Psychopathological and illness-related variables: family history of mental disorders, psychiatric comorbidities, use of alcohol and/or substance, recent trauma (total and related or not to COVID-19 pandemic), medical comorbidities, number of MDE including the current one, duration of illness, number of previous hospitalizations for MDD, suicidal ideation and/or attempt at the admission, the severity of the current MDE (mild, moderate, or severe, according to DSM-5 criteria), presence of psychotic features during the current MDE (DSM-5 criteria), and length of the current hospitalization.

Treatment-related variables: history of poor adherence to the psychopharmacological treatment, resistance to antidepressant treatments, psychotherapy started at least 1 month before admission, and variation of the antidepressant therapy during the studied hospitalization (an increase in the daily dose of the antidepressant or set-up of augmentation strategies).

Regarding the variable “recent trauma,” we considered as reference the Paykel Scale (Paykel et al., 1971), which includes a list of major traumatic events. We considered as recent traumas all the events listed in the Paykel Scale that occurred in the 6 months before the observed hospitalization. We also evaluated if the trauma was related to the COVID-19 pandemic. We considered both direct and indirect relationships with the COVID-19 pandemic. A history of poor adherence to the psychopharmacological treatment was considered positive if the patients and/or the caregivers living with her/him mentioned poor adherence to the antidepressant therapy before the current admission and/or if poor adherence was documented by the psychiatric services that followed the patients before the current admission. Resistance to antidepressants was defined as an inadequate response to at least two trials with two different antidepressant therapies belonging to different pharmacological classes for adequate dosage and duration (Souery et al., 1999; Schatzberg and Nemeroff, 2017).

Since the current study was performed on retrospective data, informed consent was not obtained, in compliance with the current European law (UE 2016/679). Data collection was integrated as part of the regular assessment procedure, did not influence therapeutic decisions or outcomes, and did not damage patients' rights. Furthermore, all sensitive personal data contained in the database have been previously anonymized. Though, according to the Local Research Ethics Committee (Comitato Etico Interaziendale A.O.U. Città della Salute e della Scienza di Torino, A.O. Ordine Mauriziano, A.S.L. Città di Torino), it was used with proper consent to processing personal data, subscribed from all patients hospitalized at the moment of the admission (declaration related to personal data processing in case of impossibility in obtaining informed consent, CEI 2017). The study was carried out in accordance with the Declaration of Helsinki (WMA with amendments) and the Good Clinical Practice (CPMP/ICH/135/95).

### Statistical analysis

First, we performed a descriptive analysis of the total sample and of the two groups, i.e., patients hospitalized before or after the lockdown. Second, we compared the two groups with one-way analysis of variance (ANOVA) for continuous variables and chi-square test for categorical variables. We also used the Z-test for proportions to compare single cells between the two groups when the chi-square test was performed with polychotomous categorical variables. We employed a multivariate logistic regression with a backward stepwise selection to find the variables more strictly associated with post-pandemic hospitalizations. The variables that were significantly different (*p* < 0.05) between the two groups were inserted in the regression model as independent variables. The dependent variable was the period of admission. The value 0 indicates pre-lockdown hospitalizations and the value 1 indicates post-lockdown ones. The variability of the dependent variable explained by the model was calculated with the *R*^2^ Nagelkerke index. Statistical significance was set at a *p*-value of <0.05. The statistical analysis was performed with the Statistical Package for Social Sciences (SPSS) software version 27 for Windows (IBM Corporation, Armonk, NY, USA).

## Results

During the study period, 2,084 patients were admitted to the two psychiatric units. Of those, 331 (15.9%) were discharged with a diagnosis of MDE in a MDD. Among them, 17 patients (5.1%) were not included due to poor information available from the electronic clinical database. The final sample consisted of 314 inpatients: 154 patients (49.0%) were admitted before and 160 patients (51.0%) were admitted after the beginning of the Italian lockdown. Patients admitted after the lockdown were less frequently followed by psychiatric services but were more frequently in psychotherapy when admitted to the psychiatric wards. They showed a higher rate of severe MDE, MDE with psychotic features, and suicidal ideation at admission. During the hospitalization, an increase in the antidepressant daily dose and the set-up of an augmentation therapy were more frequent in patients admitted after the lockdown. The characteristics of the whole sample and of patients admitted before or after the lockdown are shown in Table 1.

**Table 1:** Sociodemographic and clinical characteristics of the total sample (n = 314) and of patients hospitalized before (*n* = 154) or after (*n* = 160) the lockdown.

	**Total sample (*n* = 314)**	**Pre-lockdown (*n* = 154)**	**Post- lockdown (*n* = 160)**	**Pre-post lockdown comparison *p* (F/χ^2^)**
Total number of admission for DDM	323	159	164	0.148 (0.700)
**Socio-demographic and care-related variables**				
Age, mean (SD)	55.7 (16.7)	55.6 (16.2)	55.8 (16.1)	0.895 (0.018)
**Nationality**, ***N*** **(%)**				
Italian	286 (91.1)	138 (89.6)	148 (92.5)	0.399 (1.8)
European	10 (3.2)	7 (4.6)	3 (1.9)	
Extra European	18 (5.7)	9 (5.8)	9 (5.6)	
Male, *N* (%)	136 (43.3)	65 (42.2)	71 (44.4)	0.150 (0.70)
Unmarried, *N* (%)	152 (48.6)	67 (43.5)	85 (53.5)	0.078 (3.10)
Education level > 8 years, *N* (%)	176 (56.2)	78 (50.7)	98 (61.6)	0.276 (3.87)
Unemployed, *N* (%)	170 (54.1)	77 (50.0)	93 (58.1)	0.163 (1.95)
Presence of a social/familiar network, *N* (%)	230 (73.3)	107 (69.5)	123 (76.9)	0.139 (2.19)
Primary care assistance, *N* (%)	306 (97.5)	152 (98.7)	154 (96.3)	0.168 (1.90)
Followed by psychiatric services before admission, *N* (%)	196 (64.4)	106 (68.8)	90 (56.3)	0.021^*^ (5.29)
**Illness-related and psychopathological variables**				
Family history of psychiatric disorders, *N* (%)	99 (31.5)	49 (35.7)	44 (27.5)	0.784 (0.07)
Psychiatric comorbidity, *N* (%)	67 (21.3)	35 (22.7)	32 (20.0)	0.555 (0.35)
Substance/alcohol abuse, *N* (%)	38 (12.1)	22 (14.3)	16 (10.0)	0.244 (1.35)
Medical comorbidity, *N* (%)	172 (54.8)	82 (53.3)	90 (56.3)	0.593 (0.29)
Recent trauma (%)	95 (30.3)	35 (22.7)	49 (30.6)	0.114 (2.49)
Recent trauma non-COVID-19 related, *N* (%)	75 (23.9)	35 (22.7)	40 (25.0)	0.637 (0.22)
Recent trauma COVID-19 related, *N* (%)	20 (6.4)	0 (0.0)	20 (12.5)	NA
First major depressive episode, *N* (%)	66 (21.0)	32 (20.8)	34 (21.3)	0.918 (0.01)
Total major depressive episodes, mean (SD)	3.27 (2.5)	3.31 (2.3)	3.23 (2.6)	0.756 (0.10)
Length of disease in years, mean (SD)	12.34 (13.0)	12.5 (12.8)	12.2 (13.2)	0.871 (0.027)
Number of previous hospitalizations for MDD, mean (SD)	2.12 (2.1)	2.32 (2.4)	1.93 (1.8)	0.098 (2.756)
Suicidal ideation at the admission, *N* (%)	74 (23.6)	42 (27.3)	67 (41.9)	0.007^*^ (7.38)
Suicidal attempt at the admission, *N* (%)	109 (34.7)	31 (20.1)	43 (26.9)	0.159 (1.98)
**Severity of the major depressive episode at the admission**, ***N*** **(%)**				
Mild	37 (11.8)	20 (13.0)	17 (10.6)	0.038 (6.52)
Moderate	189 (60.2)	101 (65.6)	88 (55.0)	
Severe	88 (28.0)	33 (21.4)^#^	55 (34.4)^#^	
Psychotic features at the admission, *N* (%)	14 (4.5)	3 (2.0)	11 (6.9)	0.034^*^ (4.47)
Length of the current hospitalization in days, mean (SD)	10.49 (5.7)	10.14 (5.4)	10.84 (6.1)	0.274 (1.203)
**Treatment-related variables**				
History of low treatment adherence, *N* (%)	49 (15.6)	25 (16.2)	24 (15.0)	0.763 (0.091)
History of treatment-resistance (failure > 2 therapies), *N* (%)	112 (35.7)	61 (39.6)	51 (31.9)	0.153 (2.05)
Psychotherapy started before admission, *N* (%)	50 (13.4)	18 (11.7)	32 (20.0)	0.044^*^ (4.05)
Increase of the antidepressant dosage during the studied hospitalization, *N* (%)	48 (15.3)	16 (10.4)	32 (20.0)	0.018^*^ (5.59)
Augmentation therapy set up during the studied hospitalization, *N* (%)	39 (12.4)	13 (8.4)	26 (16.3)	0.036^*^ (4.39)
Drugs used as augmentation therapy, *N* (%),				0.144 (6.856)
Antipsychotic	154 (87.5)	68 (44.2)	86 (53.8)	
Antidepressant	15 (8.5)	9 (5.8)	6 (3.8)	
Lithium	2 (1.1)	0 (0.0)	2 (1.3)	
Other	5 (2.8)	4 (2.6)	1 (0.6)	

SD, standard deviation; ^*^p <0.05 in one-way ANOVA or χ^2^ test; ^#^p <0.05 in the two samples Z-test for proportions; NA, not applicable.

In the backward logistic regression model, four variables were significantly associated with post-lockdown hospitalizations: suicidal ideation (OR = 1.86; *p* = 0.016) and psychotic features (OR = 4.41; *p* = 0.029) at admission and the increase in the antidepressant daily dose (OR = 2.45; *p* = 0.009) and the employment of an augmentation therapy (OR = 2.25; *p* = 0.029) during the hospitalization analyzed. The model was significant (χ^2^ = 28.81; *p* < 0.001) and the Nagelkerke *R*^2^ index of the model was 28.8%. The results of the regression model are shown in Table 2.

**Table 2:** Logistic regression of sociodemographic and clinical variables associated with post-lockdown hospitalization.

**Variables of the models**	**OR (95% CI)**	***p***
Suicidal ideation at the admission	2.10 (1.28–3.43)	0.003^*^
Psychotic features at the admission	4.41 (1.16–16.71)	0.029^*^
Increase in the antidepressant dosage during the studied hospitalization	2.46 (1.16–5.02)	0.008^*^
Augmentation therapy set up during the studied hospitalization	2.41 (1.16–5.02)	0.019^*^
Psychotherapy started before admission	1.88 (0.98–3.60)	0.059
**General characteristics of the model**	*χ^2^*	***p***
Statistical significance of the regression model	28.81	<0.001
Nagelkerke *R^2^* index	28.8%	

The dependent variable was the period of admission. The value 0 indicated pre-lockdown hospitalizations and the value 1 post-lockdown ones. OR, odd ratio; CI, confidence interval; ^*^p <0.05.

## Discussion

To the best of our knowledge, this is the first study to assess the differences in the clinical characteristics of inpatients with MDD admitted for an MDE after the outbreak of the COVID-19 pandemic. We found that in post-lockdown hospitalizations, patients were less frequently followed by psychiatric services and showed more severe MDE with more frequent suicidal ideation and psychotic features. Moreover, the variables more strictly related to post-lockdown admissions were suicidal ideation and the presence of psychotic features at the admission and the intensification of the psychopharmacological treatment of the MDE during the hospitalization in terms of an increase of the antidepressant daily dose and employment of augmentation therapies.

Suicidal ideation was significantly associated with post-lockdown hospitalizations (OR = 2.1; *p* = 0.003) suggesting that the COVID-19 pandemic had a role in promoting the development of this kind of thoughts in acute patients with MDD admitted in a psychiatric ward. This result agrees with previous studies that found an increase in the rate of suicidal ideation after the lockdown in the general population (John et al., 2020; Gournellis and Efstathiou, 2021) and in patients admitted to emergency psychiatric services after the lockdown, regardless of the diagnosis (Ambrosetti et al., 2021; Boldrini et al., 2021). Furthermore, an increase in suicidality in terms of suicidal rate was also reported in previous epidemics of severe respiratory viral infections such as the 1918–1919 influenza in the U.S. (Wasserman, 1992) and the 2003 severe acute respiratory syndrome (SARS) epidemic in Hong Kong (Cheung et al., 2008). The link between the increase in suicidal ideation and the COVID-19 pandemic may depend on a complex interplay among different factors. First, the spread of the virus and the subsequent restrictions limited access to health services. Consequently, treatments were interrupted or discontinued, thus facilitating the deterioration in mental and physical health conditions (de Girolamo et al., 2020; Zhang et al., 2022). In the present study, we found similar results in terms of a reduced proportion of patients already taken in charge by psychiatric services before admission and a higher proportion of severe MDE and MDE with psychotic features. Therefore, reduced access to mental health services and more severe symptoms may have facilitated the onset of suicidal ideations in patients admitted after the lockdown. Second, to reduce the spread of COVID-19, strict public health measures were adopted in many countries including Italy. Such lockdowns may have increased social isolation, loneliness, personal and economic losses, all of which could increase the rate of suicidal ideation, particularly within at-risk groups such as patients with MDD (Gunnell et al., 2020; Reger et al., 2020; Moutier, 2021; Zhang et al., 2022).

MDE with psychotic features were significantly associated with post-lockdown hospitalizations (OR = 4.41; *p* = 0.029). As for suicidal ideation, we hypothesized that delay in treatment and social difficulties consequent to the pandemic may have supported the onset of more severe MDE associated with psychotic symptoms. Accordingly, in a sample of the USA general population evaluated during the lockdown, psychotic symptoms have been related to higher levels of psychiatric symptoms and to self-isolation, i.e., time since the last conversation, time since the person last left home, and smaller living space (Allé and Berntsen, 2021).

The other two significant associations with post-lockdown admissions concern the intensification of the psychopharmacological treatment of the MDE during the hospitalization in terms of an increase in the antidepressant daily dose (OR = 2.5; *p* = 0.008) and set-up of an augmentation therapy (OR = 2.4; *p* = 0.019). The higher proportion of patients admitted with severe MDE, MDE with psychotic features, and suicidal ideation may have enhanced the employment of more intense psychopharmacological treatments during the hospitalizations in the post-lockdown period. To the best of our knowledge, no data are available in the literature regarding antidepressant treatments for inpatients with MDD in relation to the COVID-19 pandemic. An increase in the use of antidepressants not specifically related to the treatment of MDD was reported after the COVID-19 outbreak both in the UK (Armitage, 2021) and in Italy (Farina et al., 2021). The increased symptom severity of the MDE that we found in the post-lockdown period might partly depend on the lower proportion of patients taken in charge by psychiatric services after the COVID-19 outbreak (nearly 12% less than in the pre-lockdown period). Indeed, the COVID-19 pandemic overwhelmed the healthcare system of many countries, nearly 20% of mental health centers were closed and 25% introduced limited opening hours during the COVID-19 emergency, an issue further exacerbated by the high percentage of healthcare workers affected by COVID-19 and by the patients' fear of contagion (Heymann and Legido-Quigley, 2022). Only urgent psychiatric interventions and mandatory treatments have remained unchanged, while all other activities have been somewhat reduced (Carpiniello et al., 2020; Liberman et al., 2022). These difficulties in maintaining or obtaining an engagement with psychiatric services could therefore have delayed the treatment of outpatients with MDD. This delay together with other socio-economic factors related to the COVID-19 pandemic might have enhanced the onset of MDE that is more difficult to treat.

This study has some limitations. First, the cross-sectional design did not allow us to observe patients' trends over time. Second, the worry of developing COVID-19 might have deterred patients with less mild or moderate depressive symptoms to go the emergency department. This may have led to a higher admission rate of patients with more severe MDE. Third, the data collection method based on the revision of clinic documentation (electronic healthcare database) does not provide some useful information. For example, the assessment of single depressive or suicidal symptoms with validated scales or of cognitive deficits related to the MDE was not possible. Moreover, the available data did not allow us to directly measure the association between the effect of the COVID-19 pandemic and the differences between pre- and post-lockdown depressive symptoms of patients with MDD admitted in a psychiatric ward for an MDE.

As a strength, to the best of our knowledge, this is the first study on the impact of the COVID-19 pandemic on inpatients with MDD. Our collection of data on hospitalizations enabled a detailed description of the sample and the explanation of a considerable part of the variance (28.8%) of the outcome variable of the regression model, i.e., post-lockdown admission. The large number of patients included in the sample, combined with a wide catchment area made up of people coming from all social and economic classes guarantee a certain degree of external validity to our findings. However, further multicenter investigations are needed to improve the actual knowledge on this topic.

In conclusion, our results suggest a greater severity of MDE in patients with MDD hospitalized in the post-lockdown period. This difference may have had a significant impact on the treatment as we found more frequent increases in antidepressant dosage and use of augmentation therapies during the hospitalization. This knowledge may be useful also for future calamities, suggesting that in these emergency contexts, patients with MDD would require more attention, resources, and intense treatments with a specific focus on suicide prevention.

## Data availability statement

The raw data supporting the conclusions of this article will be made available by the authors, without undue reservation.

## Ethics statement

Ethical review and approval was not required for the study on human participants in accordance with the local legislation and institutional requirements. Written informed consent to participate in this study was provided by the participants' legal guardian/next of kin.

## Author contributions

CB and PR: conceptualization and formal analysis. ED, MC, and CB: data curation. ED and MC: investigation. ED, CB, and PR: methodology. PR and VV: project administration and supervision. MC, BG, and CB: writing—original draft. BG, CB, PR, and VV: writing—review and editing. All authors contributed to the article and approved the submitted version.
